# Mammalian expression of virus-like particles as a proof of principle for next generation polio vaccines

**DOI:** 10.1038/s41541-020-00267-3

**Published:** 2021-01-08

**Authors:** Mohammad W. Bahar, Claudine Porta, Helen Fox, Andrew J. Macadam, Elizabeth E. Fry, David I. Stuart

**Affiliations:** 1 Division of Structural Biology, University of Oxford, The Henry Wellcome Building for Genomic Medicine, Headington, Oxford OX3 7BN UK; 2 The Pirbright Institute, Pirbright, Surrey GU24 0NF UK; 3 The National Institute for Biological Standards and Control, Potters Bar, EN6 3QG UK; 4 Diamond Light Source, Harwell Science and Innovation Campus, Didcot, OX11 0DE UK

**Keywords:** Vaccines, Virology

## Abstract

Global vaccination programs using live-attenuated oral and inactivated polio vaccine (OPV and IPV) have almost eradicated poliovirus (PV) but these vaccines or their production pose significant risk in a polio-free world. Recombinant PV virus-like particles (VLPs), lacking the viral genome, represent safe next-generation vaccines, however their production requires optimisation. Here we present an efficient mammalian expression strategy producing good yields of wild-type PV VLPs for all three serotypes and a thermostabilised variant for PV3. Whilst the wild-type VLPs were predominantly in the non-native C-antigenic form, the thermostabilised PV3 VLPs adopted the native D-antigenic conformation eliciting neutralising antibody titres equivalent to the current IPV and were indistinguishable from natural empty particles by cryo-electron microscopy with a similar stabilising lipidic pocket-factor in the VP1 β-barrel. This factor may not be available in alternative expression systems, which may require synthetic pocket-binding factors. VLPs equivalent to these mammalian expressed thermostabilized particles, represent safer non-infectious vaccine candidates for the post-eradication era.

## Introduction

**Fig. 1:**
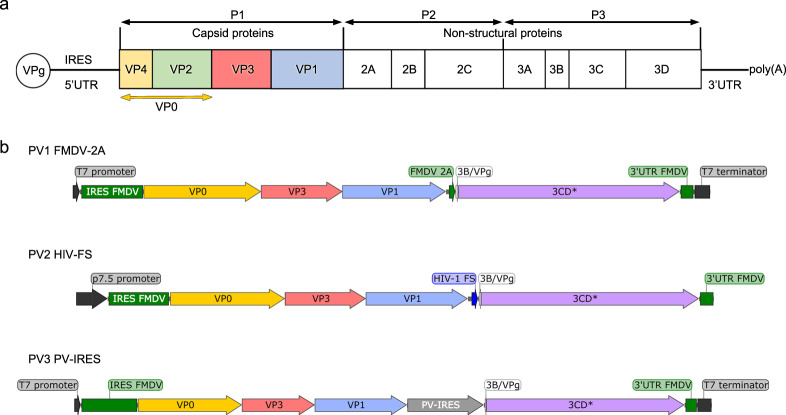
The poliovirus genome and expression cassettes designed to test P1 and 3CD* co-expression. **a** Schematic representation of the poliovirus genome highlighting the P1 region and individual capsid protein subunits generated from proteolytic processing by 3CD. **b** Format of the three separate expression cassettes used to test co-expression of P1 and 3CD* in PV1 wt (FMDV-2A), PV2 wt (HIV-FS) and PV3 wt (PV-IRES). Image was created using SnapGene® software (from GSL Biotech; available at snapgene.com).

Poliovirus (PV), belonging to the *Enterovirus* genus of the *Picornaviridae* family, is the causative agent of poliomyelitis, an acute infectious disease that can cause paralysis, mainly in young children^1^. PV has a positive-sense, single-stranded RNA genome encapsidated within a non-enveloped ~30 nm icosahedral protein capsid^2,3^. The major open reading frame (ORF) is translated as a single polyprotein comprising regions P1 (encoding the viral capsid proteins) and P2 and P3 (proteins for proteolytic processing and replication) (Fig. 1a)^2,4^. The viral protease precursor 3CD cleaves P1^5^ into the capsid proteins VP0, VP1 and VP3, and encapsidation of the viral RNA to form the mature virion is associated with cleavage of VP0 into VP2 and VP4, increasing particle stability^6,7^. Mature virions containing genome are composed of 60 copies each of the VP1-4 protomer; whilst in naturally occurring empty capsids (ECs) formed during PV morphogenesis VP0 remains uncleaved^8^. PV particles form two distinct antigenic structures: the native D-antigen associated with mature infectious virus and the non-native C-antigen^9,10^. The D-antigen elicits protective immune responses but can be converted to the C-antigenic form, for example by heating^11^. The C-antigen is conformationally expanded and does not induce long-lasting immune protection, making it unsuitable as a vaccine^11,12^.

The Global Polio Eradication Initiative (GPEI) has reduced the global incidence of wild poliovirus (WPV)^13^ such that serotypes 2 and 3 have been declared eradicated^14,15^. Both oral and inactivated polio vaccines (OPV and IPV) contributed to this success but in a future polio-free world such vaccines have disadvantages. OPV can revert to a neurovirulent wild-type (wt) phenotype, resulting in rare cases of vaccine-associated paralytic poliomyelitis (VAPP) in recipients, as well as becoming a source of circulating vaccine-derived poliovirus (cVDPV)^16^. The number of cVDPV cases now outnumbers WPV cases, having been exacerbated due to person-to-person transmission in areas with poor vaccination coverage^17^. In addition, immunodeficiency-associated vaccine derived poliovirus (iVDPV) in immune-compromised individuals contributes to the reservoir of circulating viruses, since chronic virus infection can lead to life-long virus shedding^18^. Although IPV induces effective humoral immunity that protects against poliomyelitis, it does not induce the mucosal immunity required to prevent replication of WPVs in infected individuals, and thus cannot stop continued transmission within a population^19^. Furthermore, IPV manufacture requires the growth of large quantities of live infectious PV, posing a significant risk from accidental release^20^. In order to mitigate against these bio-safety concerns there is a requirement for improved polio vaccines for the post-eradication era that do not rely on live virus for their efficacy or manufacture.

Virus-like particles (VLPs) mimic the repetitive conformation of native viral antigens but lack a genome, making them attractive as safe and potentially cheaper vaccine candidates^21,22^. Recombinant production of PV VLPs has been demonstrated in a variety of expression systems including yeast, insect, plant and mammalian cells^23–26^. An inherent problem of such recombinantly produced wt PV VLPs, like the naturally occurring ECs is that they are less stable than virus with a tendency to convert from D to C-antigenic forms^27^. However, recent progress has demonstrated that stabilisation of D-antigenic VLPs is possible^28^.

Here we show, using the modified vaccinia virus Ankara (MVA) expression system in BHK-21 mammalian cells, that by co-expression of the P1 and 3CD sequences (using different strategies to modulate 3CD levels to balance processing and toxicity)^29–31^, PV VLPs can be produced for all three wt serotypes. The most efficient strategy is then used to express a stabilised mutant for PV3^28^, which is shown to be produced in the authentic D-antigenic conformation required to induce protective immune responses. Immunisation in an experimental animal model shows this stabilised VLP induces a neutralising antibody response at least as good as IPV. High resolution structure analysis of the stabilised PV3 VLP by cryo-electron microscopy (cryo-EM) confirms that this VLP is produced in the authentic D-antigenic conformation, exhibiting features such as a bound lipid pocket factor indistinguishable from the native poliovirus. Collectively, these data suggest that recombinantly expressed stabilised PV VLPs can be a source of viable vaccine candidates in a post-eradication era.

## Results

### Molecular biology strategies for PV VLP expression using MVA vectors

To produce PV VLPs in BHK-21 cells, we tested MVA recombinants expressing P1, the structural protein precursor and 3CD, the precursor to the viral protease 3Cpro and polymerase 3Dpol (Fig. 1b). 3CD cleaves P1 more efficiently than 3Cpro^32^ and lacks polymerase activity^33^. To reduce its auto-catalytic cleavage 3CD was attenuated by introducing a cleavage site mutant, herein named 3CD*^34^.

Due to concerns about residual toxicity of 3CD*^25^, we tested three different strategies to attenuate its expression in the host cells. Each strategy was initially tried with one PV wt serotype forming a sparse matrix screen (Fig. 1b and Supplementary Fig. 1). For PV1, an FMDV-2A sequence that mediates a ‘co-translational cleavage’^35,36^ was used to link the P1 and 3CD* genes forming a single cistron for co-expression^37^. For PV2 the HIV ribosomal frameshift signal (HIV-FS)^38,39^ was placed after P1 to induce a translational frameshift, from which reinitiating translation of the downstream 3CD* ORF occurs with reduced efficiency, lowering its expression. In the case of PV3 an IRES derived from PV (PV-IRES) that has been shown to be of low efficiency in BHK-21 host cells^40^, was used to produce attenuated expression of a separate 3CD* ORF. The ORF for PV1-P1_FMDV-2A_3CD* and the P1 segment of PV3-P1_PV-IRES_3CD* were under the control of a T7 promoter and required a source of exogenous T7 RNA polymerase for initiation of transcription provided by recombinant virus MVA-T7^41^. In the PV2-P1_HIV-FS_3CD* cassette, transcription was controlled by a constitutive poxvirus p7.5 early/late promoter placed upstream of P1^42^. All cassettes contained the FMDV IRES upstream of P1 and the FMDV 3’UTR downstream of 3CD*; both the 5’ and 3’ UTR regions of FMDV participate in enhanced translation from the FMDV IRES^30^. MVA transfer vectors (Supplementary Fig. 2) with these three expression cassettes were used to generate recombinant MVA viruses called MVA-PV1_FMDV-2A, MVA-p7.5-PV2_HIV-FS and MVA-PV3_PV-IRES.

### Optimising parameters for MVA infection and expression

**Fig. 2:**
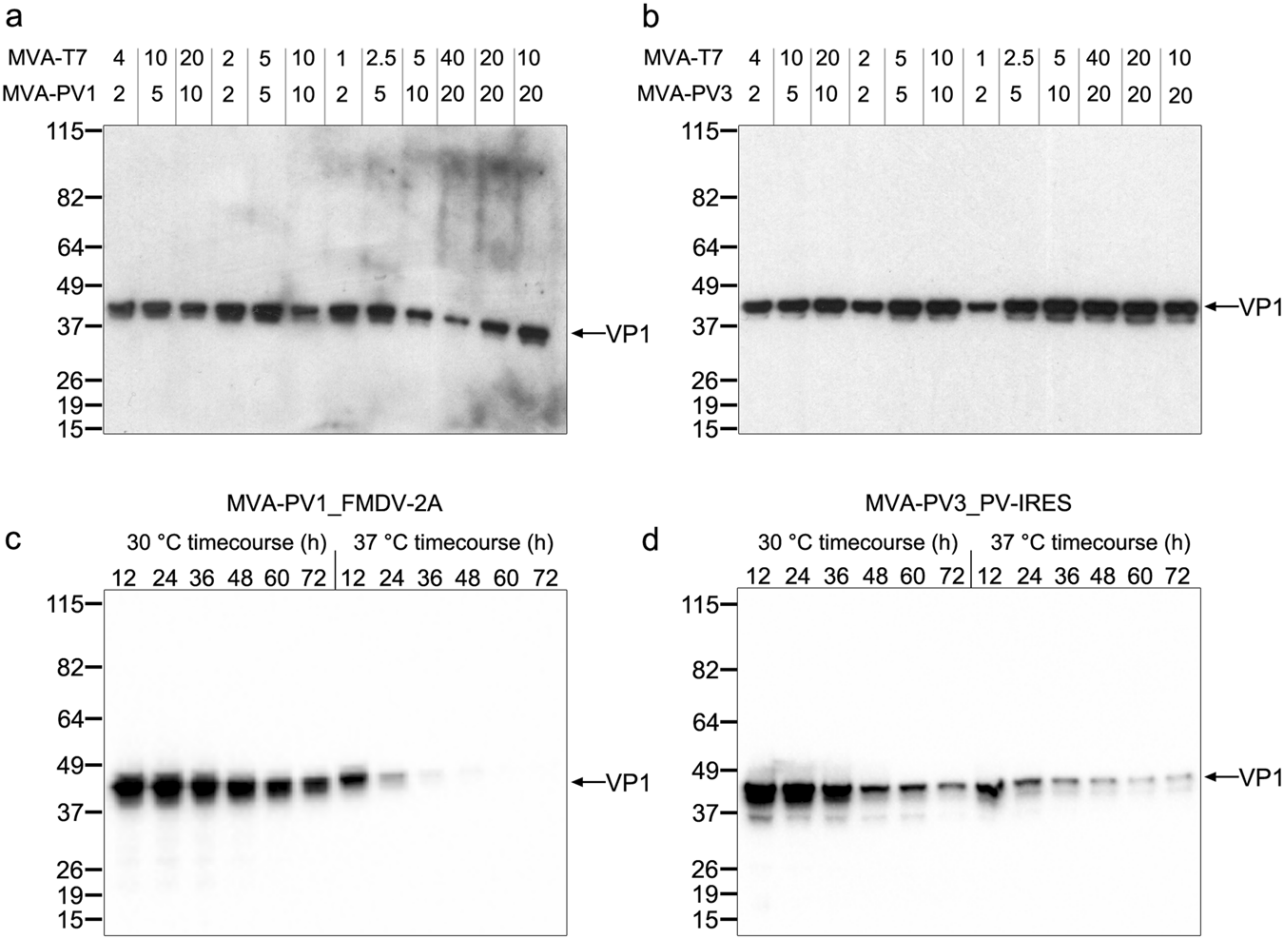
Small-scale expression of MVA-PV1 and MVA-PV3 constructs to determine optimum parameters for large-scale infection/expression. **a**, **b** Western blots of 6-well screens for MVA-PV1_FMDV-2A (**a**) and MVA-PV3_PV-IRES (**b**) co-infected with MVA-T7 to test optimum MOI ratios for expression. MOI ratios tested in each lane are shown above the blot for MVA-T7 and either MVA-PV1_FMDV-2A (MVA-PV1, **a**) or MVA-PV3_PV-IRES (MVA-PV3, **b**). **c**, **d** Western blots of MVA-PV1_FMDV-2A (**c**) and MVA-PV3_PV-IRES (**d**) co-infected with MVA-T7 at their optimum MOIs determined from (**a**) and (**b**) to test temperature (30 °C and 37 °C) and time point of harvest (12–72 h, in 12 h time points). Detection was with anti-poliovirus blend of monoclonal antibodies MAB8566 (Millipore).

In order to test initial expression and establish the optimum parameters for infection of mammalian cells with recombinant MVA-PV virus and where applicable co-infection with MVA-T7 virus, small scale experiments were performed in six-well plates of BHK-21 cells. Variables tested included multiplicity of infection (MOI), temperature and length of infection (Fig. 2).

The best MOIs for MVA-PV1 and MVA-PV3 were determined by western blot (WB) band intensity of VP1: an MOI of 5 and 10 for PV1 and PV3 expressing MVA, respectively (Fig. 2a, b) were found to be optimal. Simultaneous infection with MVA-T7^41^ was found to be required at an MOI of 5. For MVA-p7.5-PV2_HIV-FS the WB did not detect VP1 bands when the MOI was varied between 2 and 10. Based on the MOIs determined for dual infections with MVA-T7 and either MVA-PV1_FMDV-2A or MVA-PV3_PV-IRES, temperature trials at 30 and 37 °C were performed supported by reports of higher levels of protein expression at temperatures lower than 37 °C for mammalian cells^43^ (HEK cell, 33 °C). Simultaneously, incubation was monitored from 12–72 h post infection at harvest intervals of 12 h. Recombinant protein expression (estimated by VP1 band intensity on WB) was found to be highest at the lower (30 °C) temperature over the whole time course, with the earliest time point (12 h) giving the best yield for both the PV1 and PV3 expression cassettes (Fig. 2c, d). These results allowed an optimum MOI (MVA-PV1_FMDV-2A: 5, MVA-PV3_PV-IRES: 10, MVA-T7: 5), temperature (30 °C) and length of incubation for expression (12 h) to be selected for large-scale production tests.

### Matrix analysis of expression strategies

In order to verify whether the poor performance of the HIV-FS expression cassette was due to usage of this sequence or simultaneous use of constitutive promoter p7.5 in a single recombinant MVA infection mode, the PV2-P1_HIV-FS_3CD* sequence was cloned behind the T7 promoter resulting in plasmid pMVA-PV2_HIV-FS. To further compare the performances of the FMDV-2A and PV-IRES expression strategies, the matrix screen of the three PV serotypes was expanded (Table 1).

**Table 1:** Matrix of plasmids tested for transient expression by transfection into GMK cells. Expression of the P1 genes is regulated by the T7 promoter, with the T7 polymerase provided in trans through infection with MVA-T7, whilst that of the downstream 3CD gene is achieved by one of the three strategies listed on the left.

	PV1	PV2	PV3
FMDV-2A	pMVA-PV1_FMDV-2A	N.D.	pMVA-PV3_FMDV-2A
HIV-FS	N.D.	pMVA-PV2_HIV-FS	N.D.
PV-IRES	pMVA-PV1_PV-IRES	pMVA-PV2_PV-IRES	pMVA-PV3_PV-IRES

**Fig. 3:**
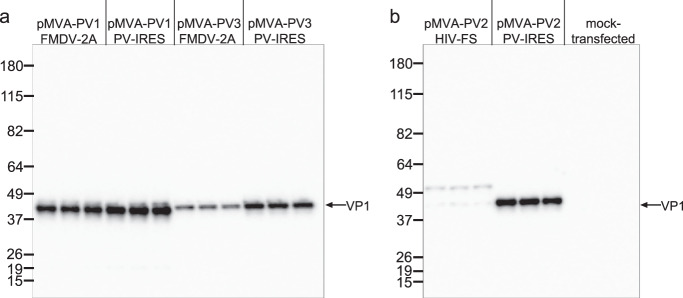
Expansion of expression screen using transient assay. **a**, **b** Western blot analysis of lysates from GMK cells infected with MVA-T7 and transfected in triplicates with pMVA-PV1_FMDV-2A, pMVA-PV1_PV-IRES, pMVA-PV3_FMDV-2A, pMVA-PV3_PV-IRES (panel **a**) and pMVA-PV2_HIV-FS, pMVA-PV2_PV-IRES or mock-transfected (panel **b**). Detection was via anti-poliovirus blend of monoclonal antibodies MAB8566 (Millipore).

To expedite testing, a transient assay was adopted, in which pMVA-PV plasmids with T7 promoter driven PV expression cassettes were transfected into mammalian cells infected with MVA-T7 virus for provision of the T7 polymerase. Green Monkey kidney (GMK) cells which are semi-permissive to MVA^44^ were found to outperform BHK-21 cells for this evaluation. Optimisation of this assay revealed that highest expression levels were obtained using similar conditions of incubation temperature (30 °C) and length of time (12 h) as identified for the dual MVA infection experiments described above. Western blot analysis of cell extracts showed that the HIV-FS cassette resulted in very low levels of VP1 (33 KDa) and generated a product of higher molecular weight, which corresponds to read-through of the frameshifting sequence up to a stop codon located 54 amino acids downstream of VP1. On the contrary expression of 3CD* from PV-IRES resulted in good levels of PV2 VP1 and also surpassed usage of the FMDV-2A co-translational cleavage approach for PV3 and to a lesser extent for PV1 (Fig. 3a, b).

### Upscaling of VLP production

**Fig. 4:**
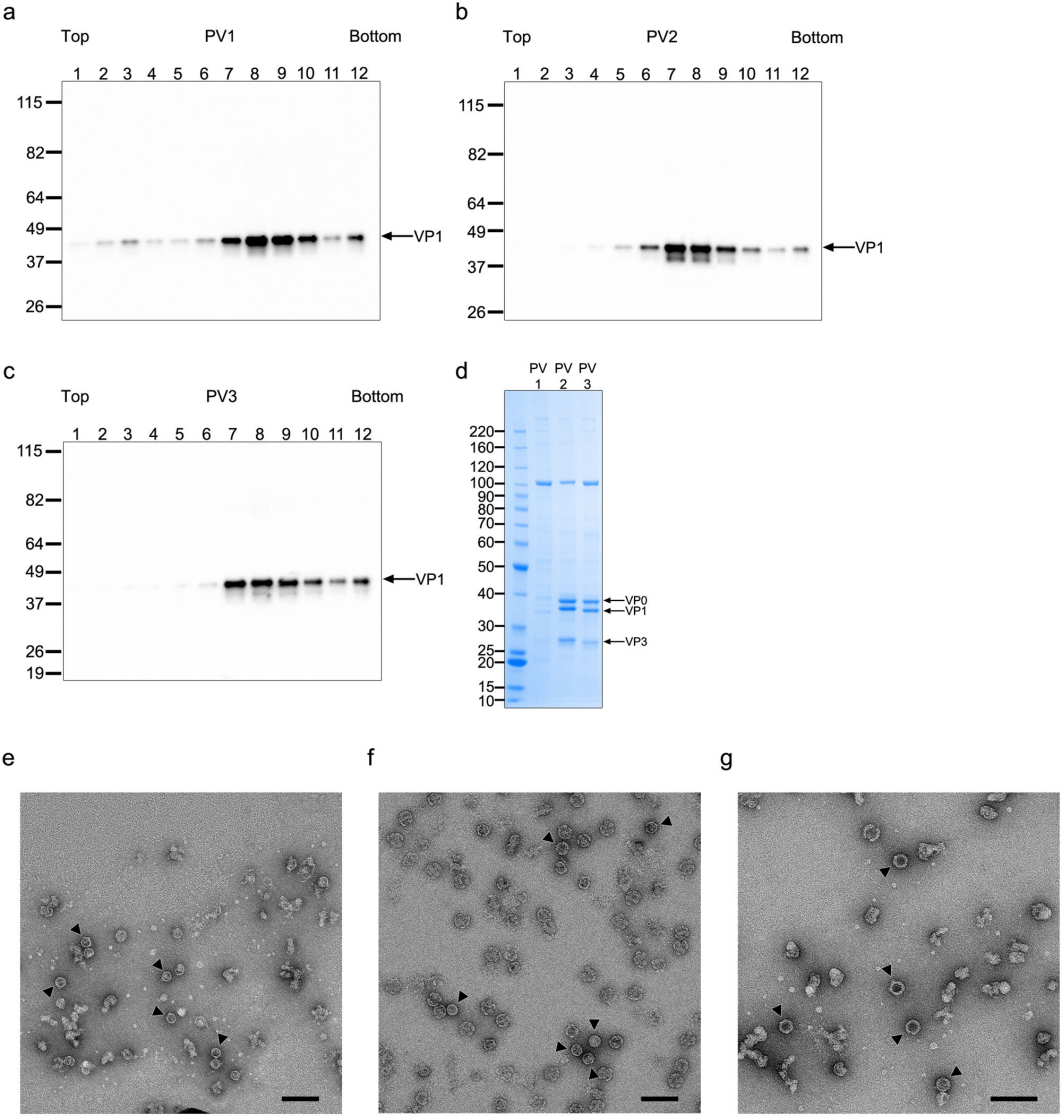
Expression and characterisation of wild-type VLPs for PV1, PV2 and PV3 produced in PV-IRES cassette. **a**–**c** Western blot of fractions from sucrose gradient purified wt VLPs of PV1 (**a**), PV2 (**b**) and PV3 (**c**) expressed using the PV-IRES cassette. Gradients are fractionated from top (fraction 1) to bottom (fraction 12). Detection was with anti-poliovirus blend of monoclonal antibodies MAB8566 (Millipore). **d** Coomassie stained gel of pooled, desalted and concentrated peak fractions for PV1 wt, PV2 wt and PV3 wt identified in **a**–**c**. Protein bands for individual subunits VP0 (37.5 kDa), VP1 (33.1 kDa) and VP3 (26.5 kDa) are labelled. **e-g** Negative stain EM images of PV1 wt, PV2 wt and PV3 wt VLPs produced from the PV-IRES expression cassette. Black arrowheads highlight individual VLPs. Scale bars 100 nm.

Following on from the transient expression screen, recombinant MVA-PV1_PV-IRES and MVA-PV2_PV-IRES viruses were generated to complement existing recombinant virus MVA-PV3_PV-IRES. Dual infections were performed alongside MVA-T7 virus in BHK-21 cells at a larger scale of 2 × 175 cm^2^ flasks, with expression at 30 °C for 12 h, followed by sucrose gradient purification of VLPs. In agreement with the findings from the transient assay, when using PV-IRES, expression was observed for all three PV serotypes (Fig. 4a-c). The PV-IRES cassette was therefore selected for the subsequent production of PV VLPs. The advantage of PV-IRES over FMDV-2A for expression of 3CD* is that an IRES allows an authentic C-terminus for the VP1 protein (the sequence of P1 is followed by a stop codon, see Supplementary Fig. 1), whereas usage of the FMDV-2A cassette leaves five-residues fused to VP1: these amino acids are derived from the VP3-VP1 cleavage site and were inserted at the VP1_FMDV-2A junction so that the 2 A sequence could be cleaved off by 3CD* (Supplementary Fig. 1).

Wild-type PV VLPs produced at the 175 cm^2^ flask scale using the PV-IRES expression format were concentrated by ultrafiltration. Analysis using SDS-PAGE and Coomassie staining showed that P1 was processed correctly into the capsid protein subunits VP0, VP3 and VP1 (Fig. 4d). Transmission electron microscopy (TEM) of negatively stained PV1 wt, PV2 wt and PV3 wt VLP samples revealed empty particles penetrated by the stain and with a distinctive icosahedral structure, ~30 nm in diameter, suggesting that the capsid protein subunits readily assembled into particles with a morphology akin to mature PV virions or naturally occurring ECs (Fig. 4e–g). Some broken and irregular shaped particles were also observed suggesting that the wt VLPs were unstable in the absence of any packaged genome (Fig. 4e–g).

### Antigenicity analysis of PV wt VLPs

**Fig. 5:**
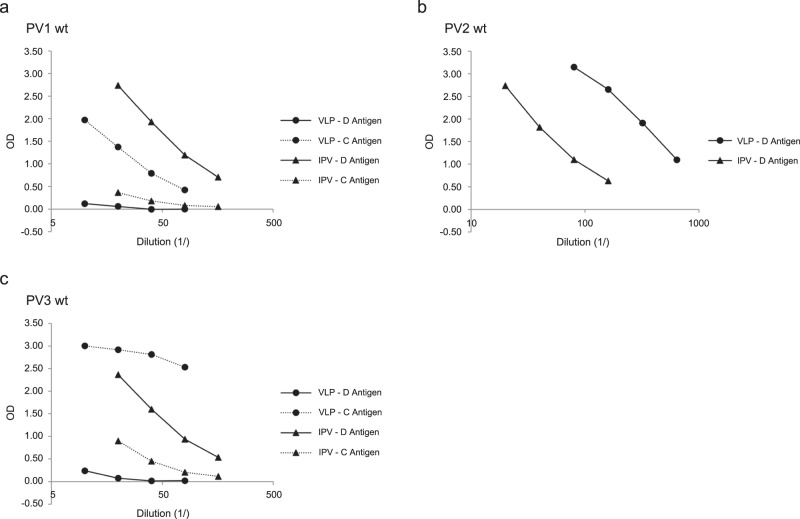
The antigenicity profile of wt VLPs for PV1, PV2 and PV3 as assessed by ELISA using monoclonal antibodies specific for either native (D) or non-native (C) conformations. **a**–**c** D and C-antigenicity of PV1 wt, PV2 wt and PV3 wt VLPs and IPV control respectively. Monoclonal antibodies used for detection were: 234 for PV1, 1050 for PV2 and 520 for PV3 (D-antigen specific) and 1588 for PV1 and 517 for PV3 (C-antigen specific). There was no C-antigen specific antibody available for PV2 wt.

Levels of native (D) and non-native (C) antigenicity in purified PV VLPs were analysed by ELISA. Both PV1 wt and PV3 wt VLPs showed very little D-antigen and were predominantly C-antigenic (Fig. 5a, c). Interestingly PV2 wt VLPs displayed D-antigenicity, quantitated by reference to IPV as a total of 600 D-antigenic (D-Ag) units, but C-antigen levels could not be analysed due to lack of an antibody specific for the non-native form (Fig. 5b). However, upon heating, the PV2 wt VLPs showed very poor antigenic stability, with the loss of 50% D-antigenicity occurring at a temperature of 34 °C (Supplementary Fig. 3)

### Stabilised PV3 SC8 VLP has native antigenicity and induces a potent immune response

**Fig. 6:**
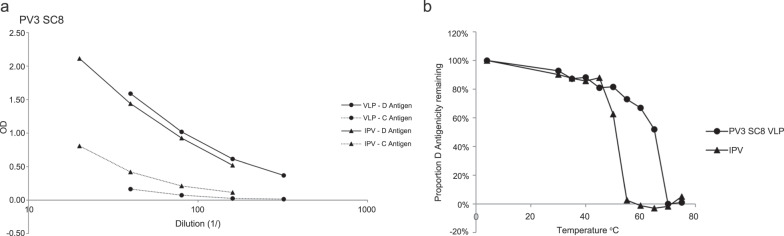
Antigenicity and thermostability of the stabilised PV3 SC8 VLP. **a** D or C-antigen reactivity of PV3 SC8 VLP or IPV assessed by ELISA. **b** Reactivity of PV3 SC8 VLP and IPV aliquots to D-antigen specific MAb 520 in ELISA after incubation at different temperatures for 10 minutes, normalised to corresponding aliquot incubated at 4 °C.

Specific combinations of amino acid mutations that enhance the antigenic thermostability of PV ECs for each of the three serotypes have been proposed^28^. We generated PV3 SC8, containing eight stabilising mutations distributed across the VP0, VP1 and VP3 capsid proteins in the PV-IRES expression cassette in shuttle vector pMVA-PV3 SC8_PV-IRES. Derived MVA recombinant MVA-PV3 SC8_PV-IRES was co-infected with MVA-T7 in 8 × 175 cm^2^ flasks of BHK-21 cells, harvested after 12 h and PV3 SC8 VLPs were sucrose gradient purified. Analysis by ELISA showed that PV3 SC8 displayed native D-antigenicity; quantitation by reference to IPV allowed to calculate that a total of 1396 D-Ag units, equivalent to 50 human doses had been produced (Fig. 6a). Conversely very little non-native C-antigenic content was present (Fig. 6a). The thermostability of PV3 SC8 was compared with PV3 IPV. As shown in Fig. 6b, the PV3 SC8 VLP shows significantly improved D-antigenic thermostability, maintaining 50% D-antigenicity at temperatures exceeding 60 °C.

**Fig. 7:**
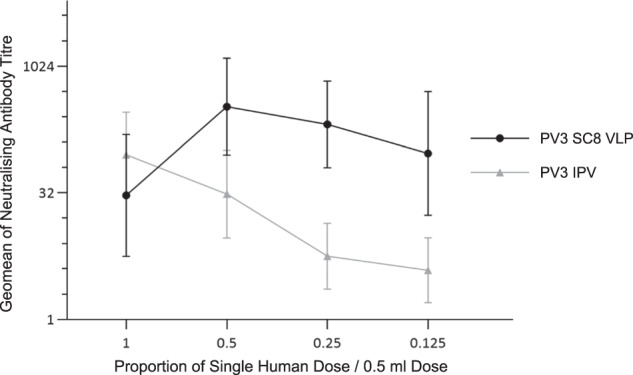
Immunogenicity of stabilised PV3 SC8 VLPs in Rats. Geometric mean neutralising antibody titres of sera from rats inoculated with a dilution series of PV3 SC8 VLPs or IPV. Bars indicate geometric standard deviation.

Rats were immunised intramuscularly (i.m.) with equivalent amounts (in D-Ag units) of stabilised PV3 SC8 VLPs or IPV reference preparation. A range of dilutions was chosen based on the regulatory assay for the batch release of commercial IPV^45^, starting at 28 D-Ag units for the type 3 component of IPV (corresponding to a single human dose). The geometric mean of neutralising antibody titres of rats immunised with PV3 SC8 VLP were higher than those obtained with IPV at all doses except the highest tested (Fig. 7). For IPV batch release, the seroconversion of each individual immunised animal is assessed using a cut-off neutralising antibody titre^46^. For this test, the neutralising antibody titre cut-off was assigned as >4. Using this assessment the data again showed superior immunogenicity of the VLPs (Table 2); a higher proportion of animals seroconverted when immunised with stabilised PV3 SC8 VLPs than with IPV, for all doses except the highest tested.

**Table 2:** Seroconversion in rats immunised (i.m.) with PV3 SC8 VLP or IPV.

	No. animals responding with an endpoint neutralising antibody titre of >4	
Proportion of single human dose	PV3 SC8 VLP	PV3 IPV
1	8/10	10/10
0.5	10/10	8/10
0.25	10/10	5/10
0.125	8/10	4/10

### Cryo-EM structure analysis of PV3 SC8 VLP

**Fig. 8:**
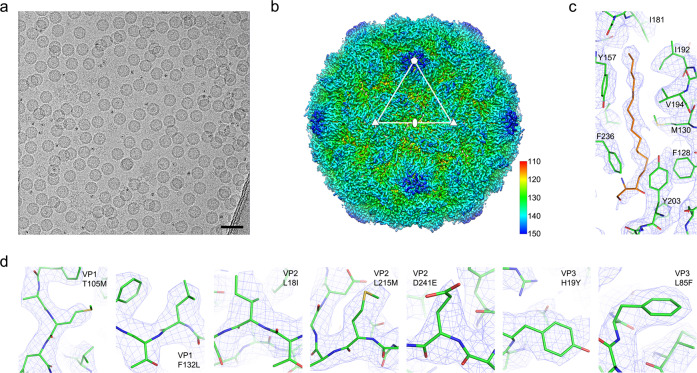
Cryo-EM structure analysis of the stabilised PV3 SC8 VLP. **a** Cryo-electron micrograph of PV3 SC8 VLPs embedded in vitreous ice. Scale bar 50 nm. **b** Cryo-EM map of the PV3 SC8 reconstruction coloured by radius from red to blue according to the scale bar shown. Representative 5-fold, 3-fold and 2-fold axes are indicated. The outer diameter of the particle is ~300 Å. **c** Electron potential map for the PV3 SC8 VP1 pocket showing the bound sphingosine-like molecule (orange) and surrounding residues of VP1 (green); key residues interacting with the modelled sphingosine are labelled. Electron potential is contoured at 1.0 σ and rendered at a radius of 2 Å around atoms. **d** Individual panels showing the cryo-EM density for the stabilising mutations introduced into the PV3 SC8 VLP. Residue numbering is equivalent to the mature capsid proteins VP1, VP2 and VP3. VP4 T67A was disordered. Electron potential is contoured at 1.0 σ and rendered at a radius of 2 Å around atoms.

The stabilised PV3 SC8 VLPs were further analysed by cryo-EM. Micrographs showed abundant, well-ordered particles with icosahedral morphology and diameter ~30 nm (Fig. 8a). A total of 9,630 ‘best’ particles from 1,465 micrographs were subjected to three-dimensional (3D) reconstruction, yielding a structure to an overall resolution of 3.0 Å (Fig. 8b), as judged by a Fourier shell correlation (FSC) cut-off of 0.143 (Supplementary Fig. 4). The bulk of the backbone polypeptide and sidechains for the VP0, VP1 and VP3 proteins were observed. The previously determined structure of PV3 SC8 VLPs from a plant expression system^25^ was fitted into the cryo-EM electron potential map, and the structure was refined and validated using standard X-ray crystallographic metrics (Supplementary Table 1a, b). The structure for the MVA PV3 SC8 protomer is in close agreement with that previously determined for PV3 SC8 produced in plants and the X-ray crystal structure of the closely related Sabin strain of PV3 wt (PDB 1PVC [10.2210/pdb1PVC/pdb]) (r.m.s.d in Cα atoms 1.32 and 0.97 Å, respectively). The MVA PV3 SC8 capsid was clearly in the D-antigenic conformation due to the absence of openings usually seen in the expanded state^12^ and the overall particle diameter of ~300 Å. The hydrophobic pocket of the VP1 capsid protein subunit was in the open state and unambiguous cryo-EM density was observed for a long-chain fatty acid molecule of ~18 carbon length bound within (Fig. 8c), which likely corresponds to the sphingosine observed bound in the native virus^47^. This would appear to correlate with the observed stability of this variant. Disordered regions corresponding to those frequently reported for natural ECs were observed e.g. the N-terminus of VP1 from residues 1 to 65, and the N-terminus of VP0 until residue 82; including the entire region of polypeptide that would correspond to the mature VP4 protein in PV. Seven of the eight stabilising mutations were clearly distinguished in the density map (Fig. 8d), the unobserved mutation (equivalent to T67A in VP4) being located in a disordered region of VP0.

## Discussion

Recombinantly produced PV VLPs offer a safe and effective means of maintaining polio vaccine supply after eradication of poliomyelitis is concluded. Expression technology to produce PV VLPs will need to meet the needs of large-scale industrial production and be of appropriate quality to induce effective immune responses in recipients. Mammalian expression has the advantage of providing an environment similar to WPV hosts, thereby producing recombinant proteins authentic to the native antigens and we therefore consider it to be a standard against which to assess expression in other systems. MVA can accommodate large inserts into the viral genome^48^, express foreign genes at high levels^49^ and since it is non-permissive in many mammalian cells, it is considered inherently safe to use under standard laboratory conditions^50,51^.

Here, we have sought to build on earlier work^26^ to demonstrate that recombinant PV VLPs can be produced in mammalian cells using the MVA expression system through co-expression of the PV capsid protein precursor P1 and 3CD protease, and used a range of expression cassettes to test the optimum strategy for balancing P1 expression and attenuating 3CD toxicity. The PV-IRES cassette utilised an inherently less toxic, uncleavable form of the protease, 3CD* cloned behind the PV-IRES to both down-regulate 3CD* expression and allow synthesis of an authentic P1 product from the T7 promoter. In the case of PV3 SC8, 175 D-Ag units i.e. just over 6 human doses were produced per 175 cm^2^ flask of BHK-21 cells (700 D-Ag units/100 ml cell culture). Indeed, efficient processing of P1 into VP0, VP1 and VP3 capsid protein subunits was observed by SDS-PAGE with negative stain TEM confirming that MVA recombinantly produced wt VLPs assemble with the same morphology as PV.

However, as has been observed for natural ECs^27^ the antigenicity of wt VLPs is mostly non-native (C-antigenic). Surprisingly, we were able to produce good yields of PV2 wt D-antigenic VLPs (note that the level of C-antigenic particles could not be quantified as there was no C-specific MAb), however, this D-antigenic content was lost upon even modest heating, reflecting the thermolability of these wt capsids. In contrast the genetically stabilised PV3 SC8 VLP was produced with high levels of D-Ag, very little C-Ag, had enhanced D-antigenic thermostability compared to IPV, and induced a neutralising antibody immune response at least as good as that from the current IPV vaccine.

Cryo-EM analysis of the stabilised PV3 SC8 VLP shows that the VP1 pocket at the bottom of the canyon that circumscribes each of the fivefold vertices of the particles is occupied by a lipidic component, modelled as a sphingosine in this study, closely resembling that seen in mature virions and natural ECs^52^. This factor was not visualised in plant produced VLPs of this same mutant^25^, possibly due to the different levels of such lipids in the plant expression system, so that sub-optimal pocket factors might have been selected. Indeed, the VP1 pocket in the plant produced PV3 SC8 VLP was readily occupied by synthetic pocket binding compounds, displacing any plant cell factors^25^. In contrast in the MVA expressed PV3 SC8 VLP, the pocket factor is observed at high occupancy, suggesting that the mammalian cell environment is beneficial for the synthesis of authentic particles like those produced by WPV infection of its host cells. Production of PV VLPs in expression systems alternative to mammalian cells may require addition of pocket binding compounds to compensate for low affinity pocket factors and ensure assembly of native conformation antigen.

This study provides proof of principle that PV VLPs can be generated in a recombinant mammalian expression system for all three serotypes of PV, and for one of these serotypes (Type 3, Saukett strain), a stabilised VLP can be produced, in the D-antigenic form necessary for vaccine candidates. The fact that these recombinant VLPs are immunologically effective, while being non-infectious further enhances their utility as alternative vaccines for PV in the post-eradication era. We envisage that similar technical approaches to those described here can be applied to VLP vaccine production for other picornaviruses and other virus families more generally.

## Methods

### Design and construction of expression cassettes and MVA transfer vectors

The P1 sequences for PV1 (Mahoney strain), PV2 (MEF-1 strain), PV3 (Saukett strain) and the 3 CD sequence (PV1 Mahoney strain) were mammalian codon optimised and synthesised de novo (GeneArt). The 3 CD sequence contained a mutation at the cleavage site between 3C and 3D: a Ser was inserted after residue 181 of 3C, which prevents self-cleavage of 3CD into 3Cpro and 3Dpol to form 3CD*^34^.

Expression cassettes were designed for each serotype, comprised of the coding sequence for P1 and 3CD*, separated either by a 2 A sequence derived from FMDV that mediates a co-translational cleavage (FMDV-2A), the HIV-1 programmed −1 ribosomal frameshifting sequence that regulates expression of the pol ORF frameshift (HIV-FS) or a PV3 Leon strain derived internal ribosome entry site (PV-IRES). Silent mutations were used for the introduction of internal restriction sites allowing the substitution of individual components of the expression cassettes and facilitating mutagenesis of P1.

The MVA transfer vector, pMVA-GFP2 (p434), containing a green fluorescent protein (GPF) gene driven by the Fowlpox virus FP4b promoter, flanked by MVA thymidine kinase gene fragments (TK)^53^ was modified by PCR to generate two intermediate cloning vectors: the p7.5 promoter was either removed and replaced with a BglII site (pMVA-GFP2_BglII) or followed by a BamHI site (pMVA-GFP2_BamHI).

A synthetic cassette encoding PV1 P1 and 3CD* sequences separated by a FMDV 2 A sequence was cloned using BstEII and NdeI sites into the vaccinia virus transfer vector pBG200-A22 in order to append the following additional elements: an FMDV IRES upstream of P1, the FMDV 3’UTR downstream of 3CD* followed by a 20 nucleotide long polyA tail, as well as the T7 promoter and terminator elements^30,37^. From the resulting plasmid pBG200-PV1 a cassette encoding T7 promoter_FMDV-5’UTR_PV1-P1_FMDV-2A_PV1-3CD*_FMDV-3’UTR_T7 terminator was excised with BglII and cloned into pMVA-GFP2_BglII to produce transfer vector pMVA-PV1_FMDV-2A.

Plasmid pBG200-PV1 was also used for replacement of the BstEII-RsrII fragment encoding PV1-P1_FMDV-2A by a synthetic fragment encoding PV2-P1_HIV-FS to produce plasmid pBG200-PV2. The BamHI fragment of pBG200-PV2 encoding FMDV-5’UTR_PV2-P1_HIV-FS_PV1-3CD*_FMDV-3’UTR was inserted downstream of the p7.5 promoter in pMVA-GFP2_BamHI to produce transfer vector pMVA-p7.5-PV2_HIV-FS.

A synthetic BstEII-RsrII fragment encoding PV3-P1_PV-IRES was directly substituted into pMVA-PV1_FMDV-2A to produce pMVA-PV3_PV-IRES.

### Construction of swapped expression cassettes and the PV3 SC8 stabilised mutant

In order to build cassettes for wild-type PV1 and PV2 in which 3CD* is expressed from PV-IRES, the P1 sequences from pMVA-PV1_FMDV-2A and pMVA-p7.5-PV2_HIV-FS were cloned into the pMVA-PV3_PV-IRES transfer vector by substitution of a BstEII-SnaBI fragment, forming plasmids pMVA-PV1_PV-IRES and pMVA-PV2_PV-IRES respectively.

To produce pMVA-PV2_HIV-FS, in which expression of the cassette is driven by the T7 promoter (instead of MVA promoter p7.5), the BstEII-RsrII fragment from pMVA-p7.5-PV2_HIV-FS comprising the PV2-P1_HIV-FS sequence was substituted into pMVA-PV1_FMDV-2A.

To generate pMVA-PV3_FMDV-2A, the BstEII-SnaBI fragment encoding PV3-P1 was excised from pMVA-PV3_PV-IRES and substituted into pMVA-PV1_FMDV-2A.

The P1 sequence for the previously reported PV3 SC8 stabilised mutant^28^ was mammalian codon optimised and synthesised de novo (GeneArt). This sequence was cloned into pMVA-PV3_PV-IRES as a BstEII-SnaBI fragment to form transfer vector pMVA-PV3 SC8_PV-IRES.

### Generation and selection of MVA recombinants

MVA recombinant viruses were produced as a service by the Jenner Institute Viral Vector Core facility (Jenner Institute, Oxford). Briefly, recombination was carried out in chicken fibroblast cell line DF-1 infected with parental MVA expressing red fluorescent protein (RFP) and transfected with an appropriate pMVA-GFP2-based plasmid encoding a PV expression cassette. Following single cell sorting by MoFlo, recombinant virus was cultured in primary chicken embryo fibroblasts (CEF). Screening by PCR was carried out through successive rounds of plaque picking to confirm identity (presence of the PV sequences) and test for purity (lack of parental MVA-RFP).

Amplification of viral stocks was in five 175 cm^2^ flasks of CEF cells harvested after 3–5 days.

MVA recombinant viruses were titrated by incubation with serial dilutions on chicken cell line DF-1. Plaque detection was either by GFP fluorescence for the MVA-PV recombinant viruses which were derived from transfer vector pMVA-GFP2 or by immunodetection with a rabbit anti-vaccinia virus polyclonal antibody (ab35219, Abcam) in the case of MVA-T7.

### Transient assay

The method was adapted from that described by Fuerst et al.^41^. BHK-21 or GMK cells were seeded into 6-well plates and after reaching a confluence of 75% were infected with MVA-T7 at an MOI of 10 in 0.5 ml DMEM supplemented with 2% FCS. GMK cells were a gift from M. Lindberg (Linnaeus University, Sweden) who had obtained them from the ATCC. BHK-21 cells were provided by the Pirbright Institute. Incubation was for 1 h at 37 °C. Meanwhile 8 µl Lipofectamine 2000 (Invitrogen) were mixed with 192 µl OptiMEM (Gibco) for 45 min at room temperature and then mixed with 2 μg of plasmid DNA in sterile water followed by further incubation at room temperature for 20 min. At this point the virus inoculum was pipetted out of the wells and the cells washed with serum-free medium before addition of the DNA/Lipofectamine/OptiMEM mix. After 4 h of incubation at 37 °C with gentle rocking, 2 ml of DMEM supplemented with 10% FCS were added per well. Incubation at 30 °C was for 12 h before cell harvest by scraping into the culture medium. Two rounds of centrifugation at 1,100 *g* for 10 min at 4 °C in a microcentrifuge allowed for complete removal of the supernatant. The cell pellet was resuspended into 50 µl PBS + 0.5% Igepal (CA-630, Sigma-Aldrich) and lysed for 30 min on ice. The cell lysate was clarified by centrifugation at 21,100 *g* for 5 min at 4 °C and 10 μl loaded onto an SDS-PAGE gel for analysis by western blotting.

### Expression and purification of PV VLPs from mammalian cells

BHK-21 cells grown in 175 cm^2^ flasks were infected with MVA-PV viruses and, where applicable, co-infected with MVA-T7 using optimum MOI(s). After 12 h of incubation at 30 °C adherent cells were released into the culture supernatant (25 ml) using cell scrapers. Cell suspensions were pooled into 50 ml conical tubes and subjected to three cycles of freezing at −20 °C and thawing at 4 °C to ensure cell lysis. For material from a single 50 ml conical tube (equating to 2 × 175 cm^2^ flasks) cell debris were removed by centrifugation at 929 *g* (Beckman Allegra X-15R centrifuge, SX4750 rotor) for 15 min at 4 °C. Clarified supernatants were treated with detergent (Igepal CA-630, Sigma-Aldrich) at a final concentration of 0.1% and incubated for 30 min on ice. VLPs were concentrated through a 2 ml 30% sucrose cushion made up in 1 × Dulbecco’s phosphate-buffered saline (DPBS, Gibco), 20 mM EDTA pH 7.0 (DPBS-EDTA) at 145,370 *g* in a SW32 rotor (Beckman) for 5 h at 4 °C. Supernatants were poured off and the VLP containing pellets were resuspended in 1/100 of the original culture volume at 4 °C overnight. Dissolved pellets were centrifuged at 10,000 *g* for 5 min at 4 °C to remove undissolved debris, before layering of the 1 ml clarified supernatant onto a 12 ml 15–45% sucrose gradient made in DPBS-EDTA. Gradients were spun at 75,484 *g* in a SW41 rotor (Beckman) for 22 h at 4 °C. Gradients were fractionated, and individual fractions were assessed for the presence of PV VLP proteins by western blotting or SDS-PAGE analysis.

### Protein western blot and SDS-PAGE analysis

Sucrose gradient fractions and protein extracts were analysed by electrophoresis on NuPAGE 4–12% Bis-Tris gels (Life Technologies). Western blot analyses were performed with an anti-poliovirus blend of monoclonal antibodies that reacts with PV1, 2 and 3 (Millipore MAB8566). Binding detection was performed with an anti-mouse antibody conjugated to horseradish peroxidase (Promega). Western blots were developed using Clarity Western ECL substrate (Bio-Rad) and imaged digitally (ChemiDoc, Bio-Rad). All western blots shown derive from the same experiments and were processed in parallel. Supplementary Figs. 5-7 show uncropped western blots. Purified VLP samples were analysed following SDS-PAGE by staining with InstantBlue Coomassie protein stain (Expedeon).

### Transmission electron microscopy

Three to four microliters of PV VLPs were adsorbed onto Formvar carbon-coated copper grids, washed with several drops of water and then stained with 2% (w/v) uranyl formate for 30 s^54^. Grids were examined using a FEI Tecnai T12 transmission electron microscope operated at 120 kV, and images acquired with a bottom-mounted digital camera.

### Cryo-EM sample preparation and data collection of PV3 SC8 VLP

Sucrose gradient purified fractions of the PV3 SC8 VLP preparation were pooled and desalted using Zeba Spin Desalting Columns with a 7 K molecular weight cut-off (MWCO) (Thermo Fisher Scientific) and concentrated using Amicon Ultra Centrifugal Filter Units devices (100 kDa MWCO, Merck Millipore) to a concentration of 0.9–1.5 mg/ml. Four microliters of PV3 SC8 VLPs were applied to glow-discharged Lacey carbon copper grids with ultra-thin carbon support film (Agar Scientific). After 30 s unbound sample was removed by manual blotting with filter paper. To increase the number of particles in the holes, grids were re-incubated with 4 μl of sample for 30 s, followed by mechanical blotting for 3–4 s and rapid vitrification in liquid ethane with a Vitrobot Mark IV plunge-freezing device (Thermo Fisher Scientific) operated at 4 °C and 100% relative humidity.

Cryo-EM data was collected at 300 kV with a Tecnai G2 ‘Polara’ microscope (FEI, now Thermo Fisher Scientific) equipped with an energy filter (GIF Quantum, Gatan) operating in zero-loss mode (0–20 eV energy selecting slit width) and a direct electron detector (K2 Summit, Gatan). Micrograph images were collected as movies (25 frames, each 0.2 s exposure) with −3.5 to −1.5 μm defocus in single-electron counting mode using SerialEM^55^. A calibrated magnification of 37,037× was used resulting in a sampling of 1.35 Å per pixel. Data acquisition parameters are summarised in Supplementary Table 1a.

### Image processing and single-particle analysis

Image processing and single-particle reconstruction was performed using RELION-3^56^. Individual movie frames were aligned and averaged with dose weighting using MotionCor2 in RELION to produce images compensated for electron beam-induced specimen drift^57^. Contrast transfer function (CTF) parameters were estimated using CTFFIND4 in RELION on dose-weighted micrographs^58^. Micrographs showing astigmatism or significant drift were discarded. Particle-picking was performed using crYOLO^59^ by first training the neural network on a randomly selected subset of ~400 manually picked particles from 50 micrographs covering a range of defocus values. Once trained crYOLO was used to pick the complete dataset in an automated manner, and the saved particle coordinates were then imported into RELION. Single-particle structure determination used established protocols in RELION for image classification and gold-standard refinement to prevent over-fitting^60,61^. A total of 14,021 particles were subjected to reference-free two-dimensional (2D) class averaging to discard bad particles and remove junk classes. The particle population was further enriched by 3D classification using the previously determined cryo-EM structure of PV3 SC8 from a plant expression system (Electron Microscopy Data Bank (EMDB) accession code EMD-3747 [https://www.emdataresource.org/EMD-3747])^25^ low-pass filtered to 60 Å as an initial reference. A final set of 9630 particles was selected for 3D auto-refinement with the application of icosahedral symmetry throughout. A representative class from the end of 3D classification was low pass filtered to 40 Å to avoid bias and used as a reference during refinement. After the first round of refinement the dataset was subjected to CTF refinement^62^ to estimate beam tilt, anisotropic magnification, per-particle defocus and astigmatism, and also Bayesian polishing of beam-induced motion-correction with trained parameters^56^. This procedure was performed twice with 3D auto-refinement after each round. The final resolution was estimated using a Fourier shell correlation (FSC) threshold of 0.143^60^. The map was sharpened using Post-processing in RELION by applying an inverse B-factor of −82.6 Å^2^. Local resolution was estimated using the RELION implementation of local resolution algorithm. Data processing statistics are summarised in Supplementary Table 1a.

### Atomic model building and refinement

The atomic coordinate of the previously determined structure of PV3 SC8 (PDB 5O5B [10.2210/pdb5O5B/pdb]) was manually placed into the electron potential map for the MVA expressed PV3 SC8 VLP and rigid-body fitted with UCSF Chimera^63^. The fitting was further improved with real-space refinement using Coot^64^. Manual model building was performed using the tools in Coot^64^ in combination with iterative positional and B-factor refinement in real space with Phenix^65^. Only atomic coordinates were refined; the maps were kept constant. Each round of model optimisation was guided by cross-correlation between the map and the model. Structure validation was performed using Coot^64^ and the MolProbity^66^ functions integrated within Phenix. Refinement statistics are shown in Supplementary Table 1b. Molecular graphics were generated using Pymol^67^ and UCSF Chimera^63^.

### Antigenic potency ELISA

PV VLP samples were tested for levels of D and C-antigenicity using a non-competitive sandwich ELISA^68^. Briefly, samples were diluted two-fold in assay diluent and captured with a serotype-specific polyclonal antibody. Serotype-specific, D-antigen- or C-antigen-distinctive monoclonal antibodies were used for detection, followed by anti-mouse peroxidase conjugate. Where appropriate, the D-antigen content of test samples was evaluated against an IPV reference of assigned D-antigen units by parallel line analysis using the statistical software package Combistats^69^. Monoclonal antibodies used for detection were: 234 for type 1, 1050 for type 2 and 520 for type 3 (D-antigen specific) and 1588 for type 1 and 517 for type 3 (C-antigen specific). There was no C specific type 2 antibody available.

### Thermostability analysis

PV VLPs or IPV were diluted in Dulbecco’s Phosphate Buffered Saline (Sigma D8662) and duplicate aliquots were incubated on ice (control) or at various temperatures between 30 °C and 75 °C for 10 minutes. Thermostability of the samples was then assessed by measuring loss in D-antigenicity by ELISA. For PV3, the conformational change from D to C-antigenicity could also be measured due to the availability of a C-antigen specific monoclonal antibody.

### Immunogenicity

The immunogenicity of PV VLPs was assessed in Wistar rats by a neutralising antibody assay according to the Pharmacopeial method^70^. Briefly, for each antigen 4 groups of 10 rats were inoculated i.m. (0.25 ml in each hind leg) with a dose-range comprising dilutions of a single human dose, as measured by D-Ag ELISA. After 21–22 days blood samples were collected and assayed for the presence of neutralising antibodies. Titres were compared with those obtained using a concurrent IPV reference preparation of known potency. This method has been established at NIBSC for the release of IPV batches and neutralising antibody titre cut offs have been previously assigned by the laboratory to assess seroconversion.

### Ethics declarations

All animal experiments were performed under licenses granted by the UK Home Office under the Animal (Scientific Procedures) Act 1986 revised 2013 and reviewed by the internal NIBSC Animal Welfare and Ethics Review Board. The rat immunogenicity experiments were performed under Home Office licence P856F6831.

### Reporting summary

Further information on research design is available in the Nature Research Reporting Summary linked to this article.

## Supplementary information

Supplementary Information

Reporting Summary

## Data Availability

The atomic coordinates of MVA PV3 SC8 have been submitted to the Protein Data Bank under accession code 6Z6W. The cryo-EM density map has been deposited in the Electron Microscopy Data Bank under accession code EMD-11106. The data that support the findings of this study are available from the corresponding authors on request.
