# Supplementary material for: Mammalian expression of virus-like particles as a proof of principle for next generation polio vaccines
Source: NPJ Vaccines. 2021 Jan 8;6:5. doi: 10.1038/s41541-020-00267-3 (PMC7794334; doi:10.1038/s41541-020-00267-3)

Supplementary material

Supplementary Fig. 1: Structure of the expression cassettes for 3CD*. **a** Sequence of the FMDV-2A peptide in pMVA-PV1_FMDV-2A. **b** Sequence of the HIV-1 FS sequence in pMVA-p7.5-PV2_HIV-FS. **c** Sequence of the IRES from PV3/Leon/37 (accession K01392) in pMVA-PV3_PV-IRES. Image was created using SnapGene® software (from GSL Biotech; available at snapgene.com).

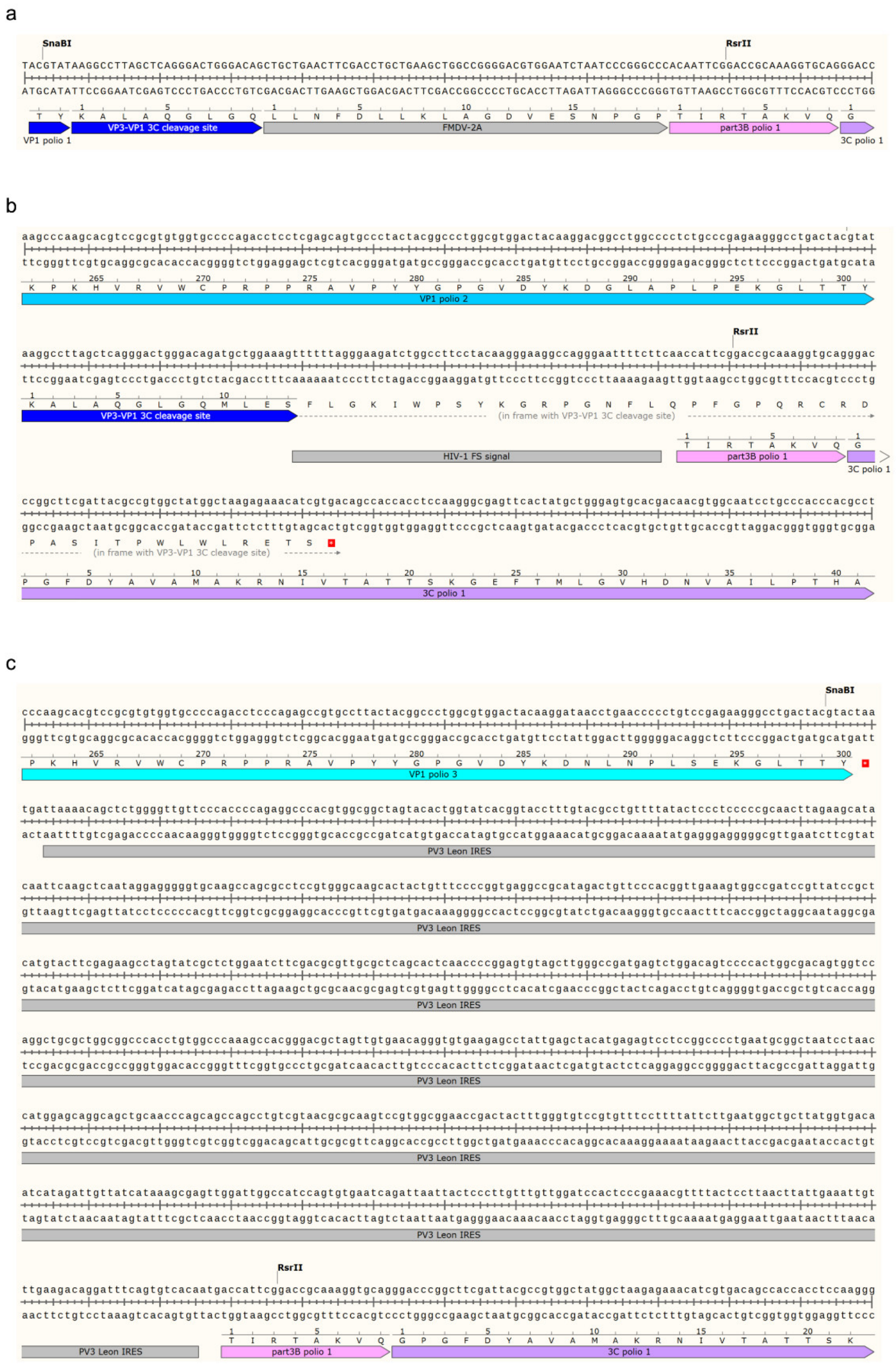
a
SnaBI
RsrII
VP1 polio 1
VP3-VP1 3C cleavage site
FMDV-2A
part3B polio 1
3C polio 1
b
VP1 polio 2
RsrII
VP3-VP1 3C cleavage site
(in frame with VP3-VP1 3C cleavage site)
HIV-1 FS signal
part3B polio 1
3C polio 1
c
SnaBI
VP1 polio 3
PV3 Leon IRES
RsrII
part3B polio 1
3C polio 1

Supplementary Fig. 2: Plasmid map of a typical pMVA transfer vector, showing the relative locations of the GFP and PV expression cassettes and the main restriction sites used for subcloning. Image was created using SnapGene® software (from GSL Biotech; available at snapgene.com).

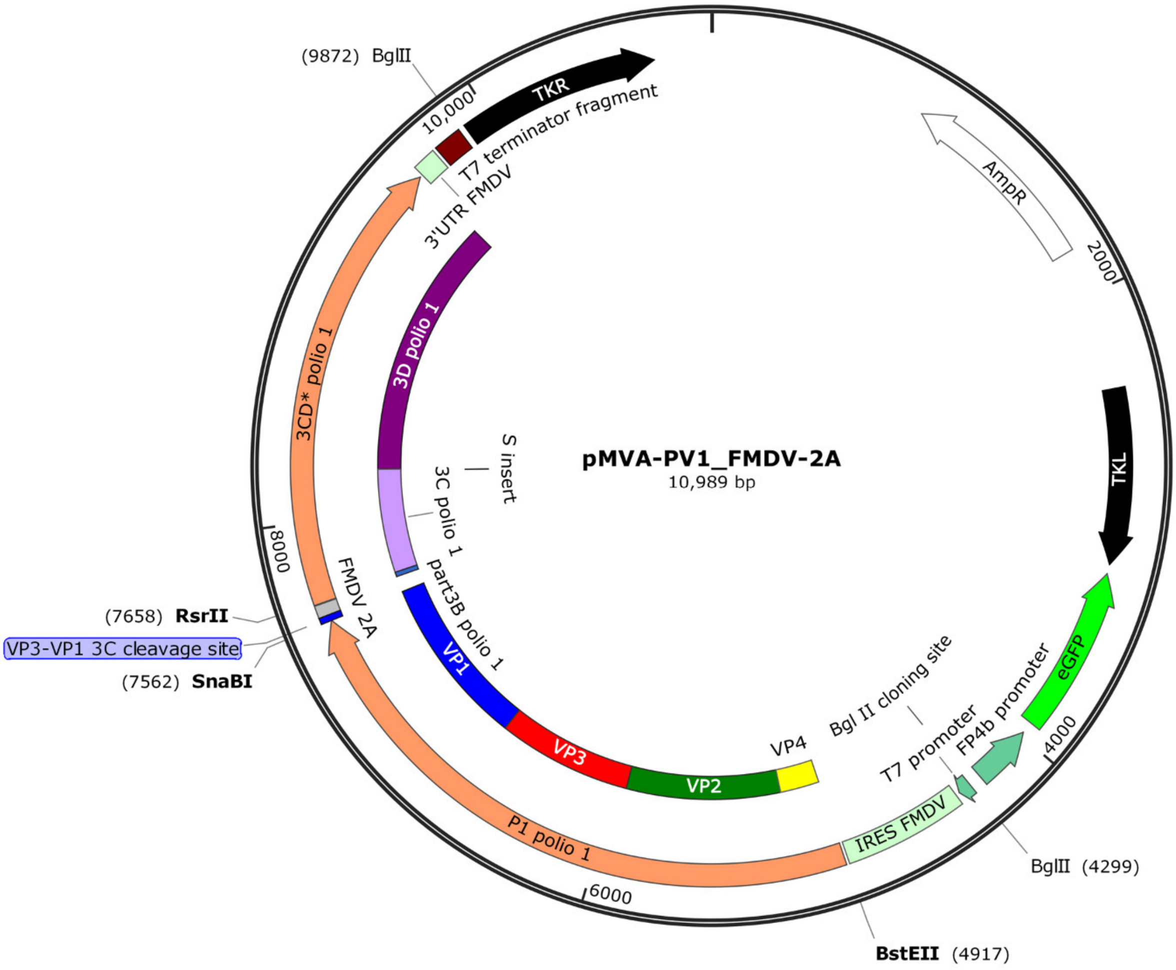

Supplementary Fig. 3: Antigenic thermostability of PV2 wt VLP. Reactivity of PV2 wt VLP and IPV aliquots to D-antigen specific MAb (1050 for PV2) in ELISA after incubation at different temperatures for 10 minutes, normalised to corresponding aliquot incubated at 4 °C.

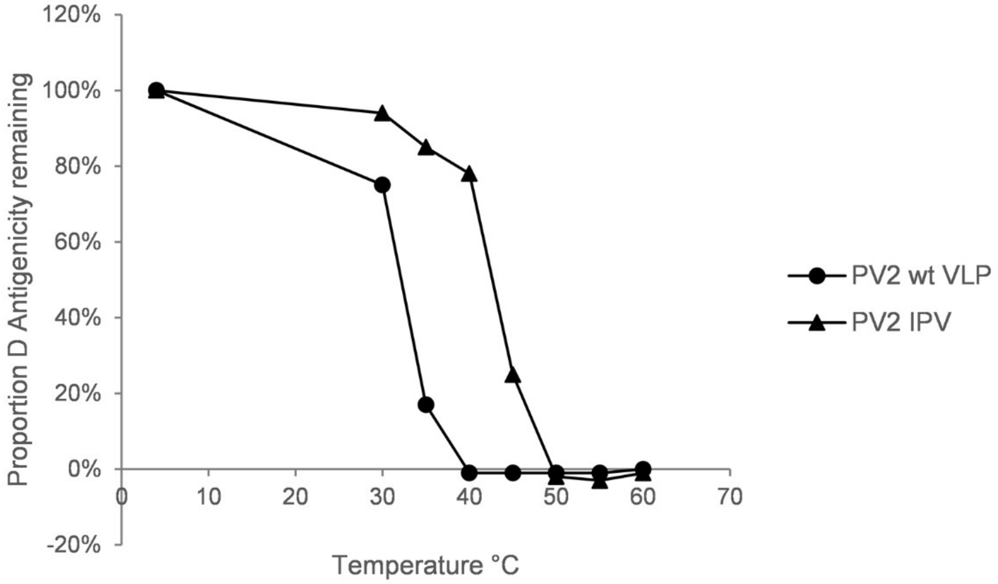

Supplementary Fig. 4: Resolution of the PV3 SC8 VLP icosahedral reconstruction. **a** Fourier shell correlation (FSC) calculated between two independent half sets of data as a function of spatial frequency is plotted. FSC is plotted for the original unmasked half-maps (grey) and masked half-maps that had density corresponding to solvent removed (blue). FSC is also shown for phase-randomized half-maps (red) used to compensate for possible effects of the masking procedure before calculating the final corrected FSC (black). Good agreement between the masked and corrected curves indicated no adverse effects from the masking. The resolution at which the corrected curve drops below the FSC=0.143 threshold is indicated with an arrow. **b** Local resolution analysis of the final PV3 SC8 cryo-EM electron potential map, as assessed by RELION local resolution estimation. A central slice through the VLP is viewed along the 2-fold axis and the distribution of local resolution is shown coloured from blue (2.9 Å) to red (3.7 Å) according to the scale bar shown.

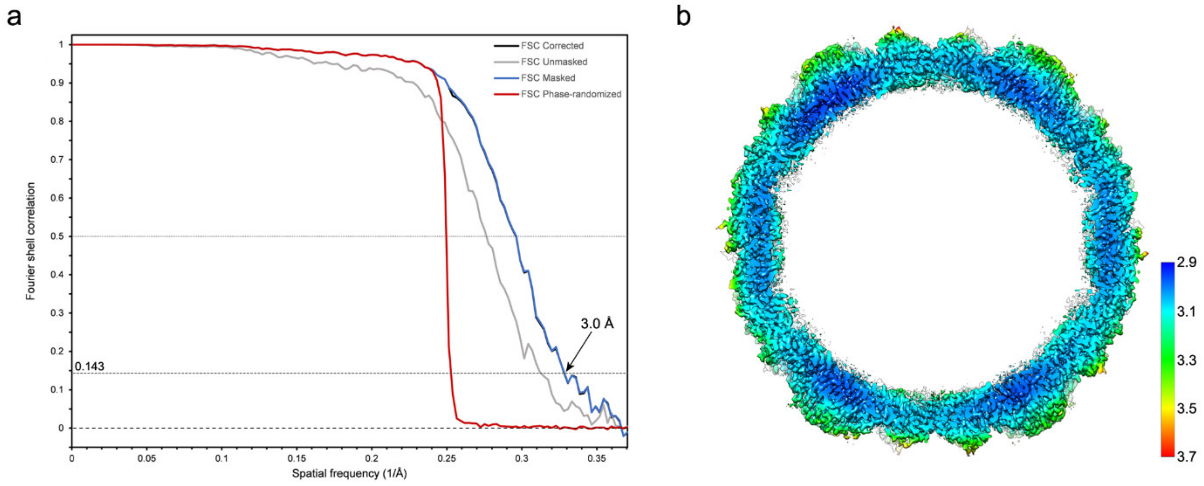

Supplementary Table 1: PV3 SC8 VLP Cryo-EM statistics.

(a) Data collection and image processing.

**Parameter**	**Value**
**Data collection**	
Voltage (kV)	300
Magnification (×)	37,037
Defocus (µm)	-3.5 to -1.5
Dose rate (e^{-}/pixels/s)	14.58
Frames	25
Frame length (s)	0.2
Total dose ($e^{-}/Å^2$)	40
Micrographs	1465
**Data processing**	
Pixel size (Å)	1.35
Particles	9630
Box size (pixels)	280
Symmetry	I1
Accuracy of rotations (°)	0.274
Accuracy of translations (Å)	0.405
Resolution (Å)	3.0
Map sharpening B-factor ($Å^2$)	-82.6

(b) PV3 SC8 VLP structure refinement and validation.

**Parameter**	**Value**
**Model composition**	
Atoms	5646
Protein	5625
Other	21
Protein residues	713
**Refinement**	
Resolution (Å)	3.0
Map CC (Mask)	0.82
Map CC (Volume)	0.72
**RMS deviations**	
Bond lengths (Å)	0.006
Bond angles (°)	0.955
**Average B-factor ($Å^2$)**	
Protein	31.56
Other	28.63
**Validation**	
Molprobity score (percentile)	5.46 (92nd)
Clashscore, all atoms (percentile)	1.43 (97th)
Ramachandran plot favoured (%)	97.29
Ramachandran plot allowed (%)	2.71
Ramachandran plot outliers (%)	0.00
Rotamer favoured (outliers) (%)	93.74 (0.80)
Cβ outliers (%)	0.00
CaBLAM outliers (%)	1.00
CA geometry outliers (%)	0.58

Supplementary Fig. 5: Full image of western blots shown in Fig. 2a-d.

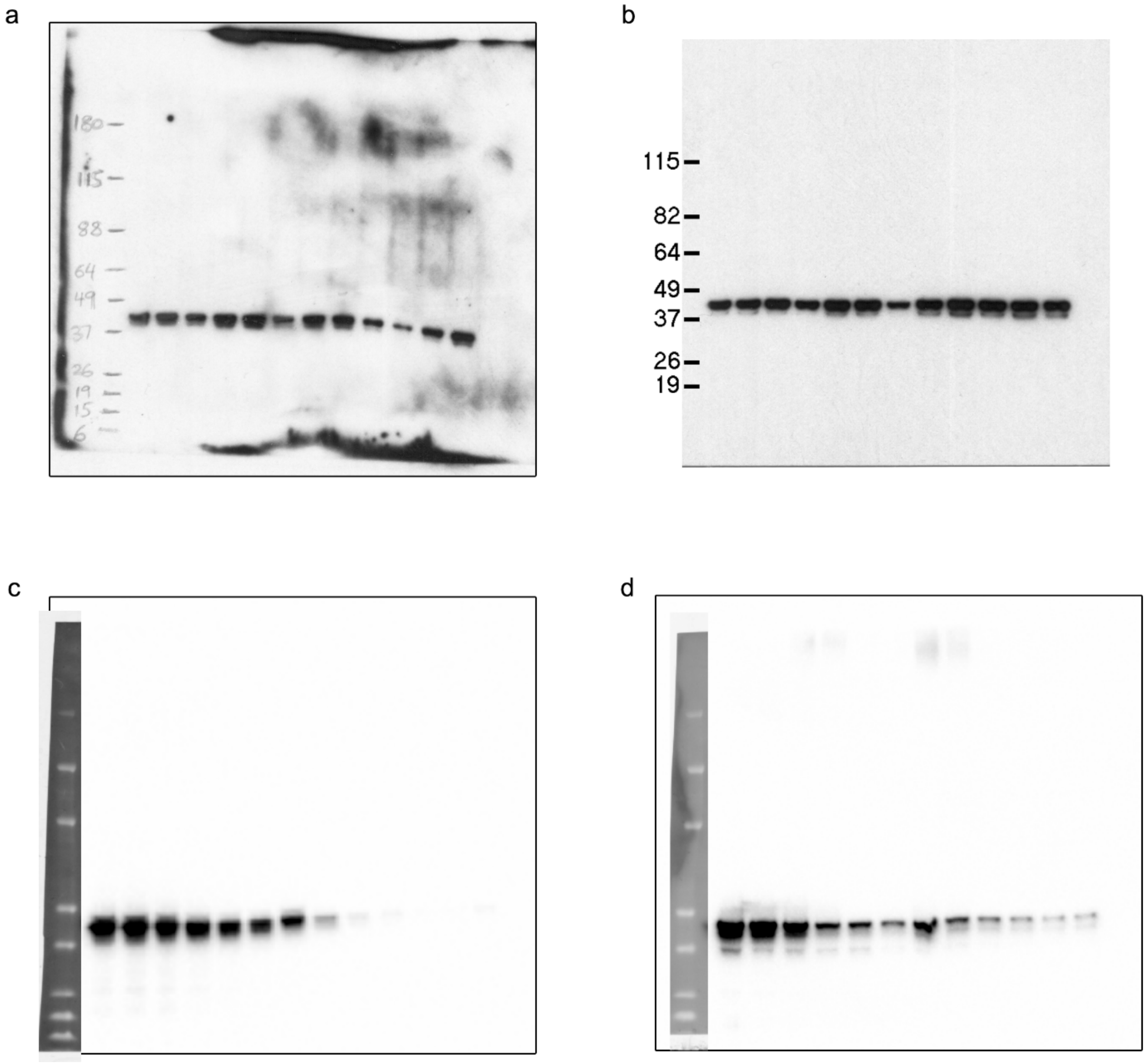

Supplementary Fig. 6: Full image of western blots shown in Fig. 3a, b.

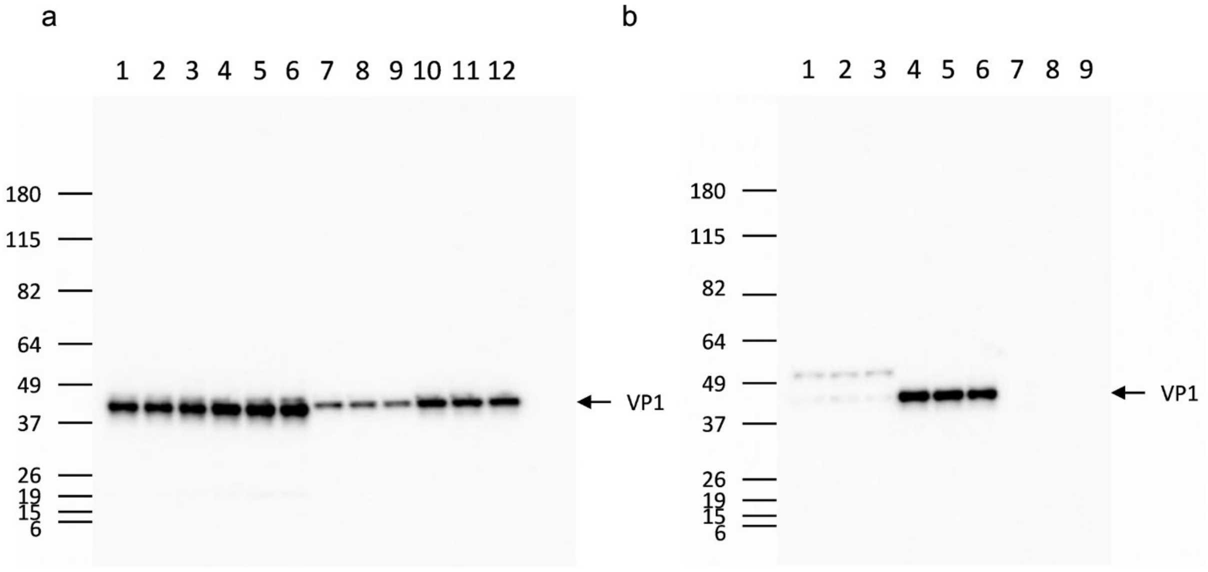

Supplementary Fig. 7: Full image of western blots shown in Fig. 4a-c.

a

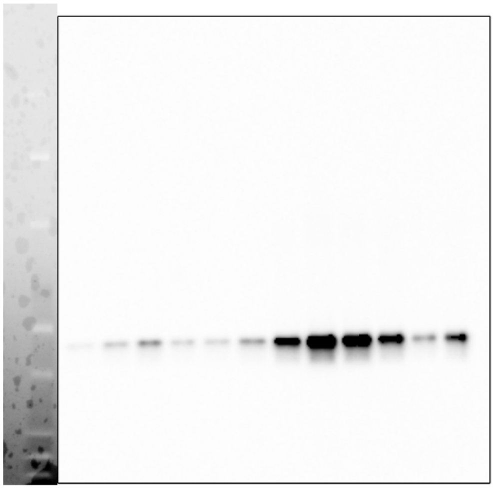

b

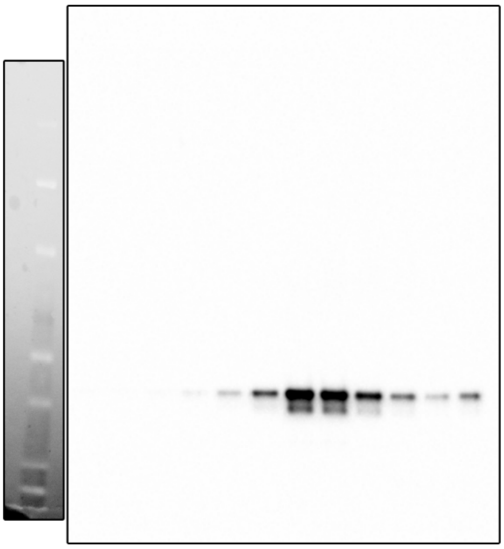

c

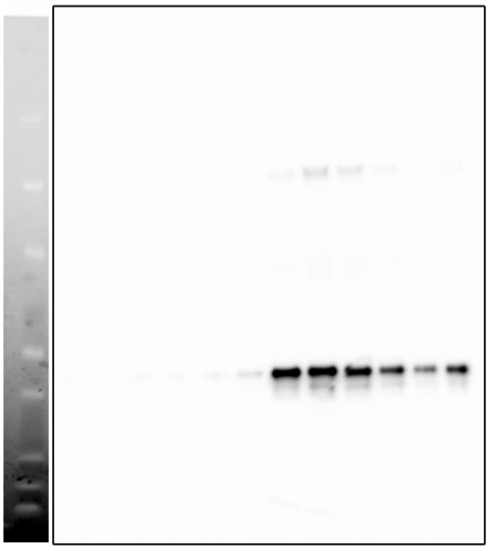

Supplement: Supplementary file 1 — Supplementary Information [file 41541_2020_267_MOESM1_ESM.pdf]
