# Supplementary material for: Mammalian expression of virus-like particles as a proof of principle for next generation polio vaccines
Source: NPJ Vaccines. 2021 Jan 8;6:5. doi: 10.1038/s41541-020-00267-3 (PMC7794334; doi:10.1038/s41541-020-00267-3)
Supplement: Supplementary file 2 — Reporting Summary [file 41541_2020_267_MOESM2_ESM.pdf]

nature research

Corresponding author(s): Mohammad W. Bahar
David I. Stuart

Last updated by author(s): Oct 5, 2020

Reporting Summary

Nature Research wishes to improve the reproducibility of the work that we publish. This form provides structure for consistency and transparency in reporting. For further information on Nature Research policies, see our Editorial Policies and the Editorial Policy Checklist.

Statistics

For all statistical analyses, confirm that the following items are present in the figure legend, table legend, main text, or Methods section.

n/a	Confirmed	
☒	☐	The exact sample size (*n*) for each experimental group/condition, given as a discrete number and unit of measurement
☒	☐	A statement on whether measurements were taken from distinct samples or whether the same sample was measured repeatedly
☐	☒	The statistical test(s) used AND whether they are one- or two-sided<br>*Only common tests should be described solely by name; describe more complex techniques in the Methods section.*
☒	☐	A description of all covariates tested
☒	☐	A description of any assumptions or corrections, such as tests of normality and adjustment for multiple comparisons
☒	☐	A full description of the statistical parameters including central tendency (e.g. means) or other basic estimates (e.g. regression coefficient) AND variation (e.g. standard deviation) or associated estimates of uncertainty (e.g. confidence intervals)
☒	☐	For null hypothesis testing, the test statistic (e.g. *F*, *t*, *r*) with confidence intervals, effect sizes, degrees of freedom and *P* value noted<br>*Give P values as exact values whenever suitable.*
☒	☐	For Bayesian analysis, information on the choice of priors and Markov chain Monte Carlo settings
☒	☐	For hierarchical and complex designs, identification of the appropriate level for tests and full reporting of outcomes
☒	☐	Estimates of effect sizes (e.g. Cohen's *d*, Pearson's *r*), indicating how they were calculated

Our web collection on statistics for biologists contains articles on many of the points above.

Software and code

Policy information about availability of computer code

Data collection	SerialEM version 3.x software for microscope control and data acquisition.
Data analysis	RELION version 3.1 for cryo-EM data processing, Coot version 0.8.9 for model building, UCSF Chimera version 1.14 for structure analysis. CombiStats version 4.0 for parallel lines analysis of data from potency assays.

For manuscripts utilizing custom algorithms or software that are central to the research but not yet described in published literature, software must be made available to editors and reviewers. We strongly encourage code deposition in a community repository (e.g. GitHub). See the Nature Research guidelines for submitting code & software for further information.

Data

Policy information about availability of data

All manuscripts must include a data availability statement. This statement should provide the following information, where applicable:

- Accession codes, unique identifiers, or web links for publicly available datasets
- A list of figures that have associated raw data
- A description of any restrictions on data availability

The atomic coordinates of MVA PV3 SC8 have been submitted to the Protein Data Bank under accession code 6Z6W. The cryo-EM density map has been deposited in the Electron Microscopy Data Bank under accession code EMD-11106. The data that support the findings of this study are available from the corresponding authors on request.

Field-specific reporting

Please select the one below that is the best fit for your research. If you are not sure, read the appropriate sections before making your selection.

☒ Life sciences ☐ Behavioural & social sciences ☐ Ecological, evolutionary & environmental sciences

For a reference copy of the document with all sections, see nature.com/documents/nr-reporting-summary-flat.pdf

Life sciences study design

All studies must disclose on these points even when the disclosure is negative.

Sample size	n/a
Data exclusions	No data excluded.
Replication	Reproducibility was verified for all experiments, e.g. experiments performed in triplicate are described as such.
Randomization	n/a
Blinding	n/a

Reporting for specific materials, systems and methods

We require information from authors about some types of materials, experimental systems and methods used in many studies. Here, indicate whether each material, system or method listed is relevant to your study. If you are not sure if a list item applies to your research, read the appropriate section before selecting a response.

Materials & experimental systems

n/a	Involved in the study	
☐	☒	Antibodies
☐	☒	Eukaryotic cell lines
☒	☐	Palaeontology and archaeology
☐	☒	Animals and other organisms
☒	☐	Human research participants
☒	☐	Clinical data
☒	☐	Dual use research of concern

Methods

n/a	Involved in the study	
☒	☐	ChIP-seq
☒	☐	Flow cytometry
☒	☐	MRI-based neuroimaging

Antibodies

Antibodies used	Anti-poliovirus blend MAB8566 (Millipore), Anti-mouse antibody conjugated to horseradish peroxidase (Promega W4021). Monoclonal antibodies used for detection were: 234 for polio type 1, 1050 for polio type 2 and 520 for polio type 3 (D-antigen specific) and 1588 for type 1 and 517 for type 3 (C-antigen specific).
Validation	MAB8566 is validated for immunofluorescence, Promega W4021 is validated for western blotting.<br>MAbs 234, 1050, 520, 1588 and 517 were validated by collaborators at NIBSC for use in detection of poliovirus capsids.

Eukaryotic cell lines

Policy information about cell lines

Cell line source(s)	CEF and BHK-21 cells were sourced from the Pirbright Institute. GMK cells were sourced from the American Type Culture Collection (ATCC). DF-1 cells were sourced from The Jenner Institute Oxford.
Authentication	n/a
Mycoplasma contamination	n/a
Commonly misidentified lines<br>(See ICLAC register)	n/a

Animals and other organisms

Policy information about studies involving animals; ARRIVE guidelines recommended for reporting animal research

Laboratory animals	Wistar rats.
Wild animals	The study did not involve wild animals.
Field-collected samples	The study did not involve samples collected from the field.
Ethics oversight	All animal experiments were performed under licenses granted by the UK Home Office under the Animal (Scientific Procedures) Act 1986 revised 2013 and reviewed by the internal NIBSC Animal Welfare and Ethics Review Board. The rat immunogenicity experiments were performed under Home Office licence P856F6831.

Note that full information on the approval of the study protocol must also be provided in the manuscript.
